# 

**Published:** 1891-05-16

**Authors:** 


PRICE ONE PENNY.

No. 242,—Vol. X.— May 16th, 1891.

[Registered at the General Post Office as a Newspaper for transmission in the United Kingdom and Abroad.]

WITH EXTRA NURSING SUPPLEMENT.

Per Annum, 6s. 6d.; post free.

Monthly Parts, 6d.; post free 8d.

Ditto per Annum, 8s., post free.

### THE HOSPITAL

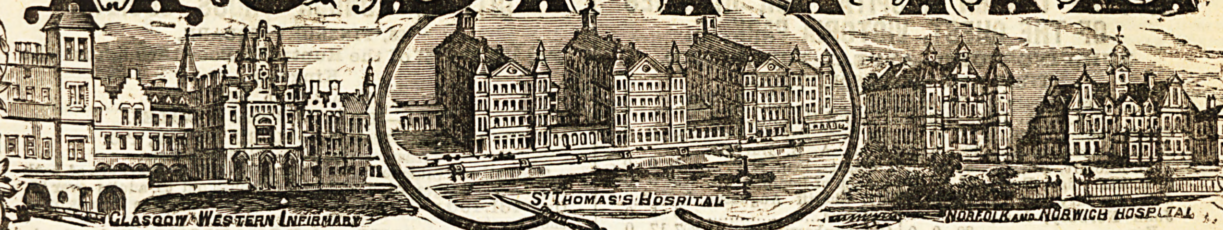

A WEEKLY JOURNAL OF

### Science, Medicine, Nursing and Philanthropy.

### SATURDAY, MAY 16th, 1891.

| | |
|---|---|
| **Suburban Doctors and Suburban Hospitals** | 73 |
| **Passing Topics.**—The Norwich Neophyte | 74 |
| **The Doctor's Armchair.**—Costly Sacrifices | 75 |
| **Vivisection Questioned** | 76 |
| **Editor's Letter-Box** | 76 |
| **Everybody's Page** | 77 |
| **Notes and News** | 78 |
| **Annotations.**—The Influenza Epidemic—The Medical Hat—A General Practitioner of the Old School | 80 |
| **General Charities—Scraps and Gleanings** | 79 |
| **The Practitioner's Mirror and Retrospect:**— | |
| MEDICINE.—Carbuncle and Furuncle—Renal Torpidity | 81 |
| SURGERY.—Ingleby Lectures: Lecture VI. | 82 |
| THERAPEUTICS.—Quebracho | 83 |
| NEW DRUGS, APPLIANCES, AND THINGS MEDICAL.—Malt Extract, "Standard" Brand | 83 |
| **Neuritis of the Peripheral Nerves** | 84 |
| **The Student of Medicine.**—Leading Athletes—Appointments—Vacancies—Pass Lists—Events for the Month. | |

### EXTRA SUPPLEMENT—THE NURSING MIRROR.

| | |
|---|---|
| **En Passant.**—Nurses for Northallerton—Another Warning—Short Items—The Countess of Zetland—The Second Thousand—Nurses' Home—Wants and Workers | xxxvii |
| **Lectures on Surgical Ward Work and Nursing.**—By ALEX MILES, M.B. Edin., C.M., F.R.C.S.E.—Lecture XXIV.—Sayre's Jacket and Starch Splints | xxxviii |
| **Work in the Holy Land** | xxxix |
| **Death in Our Ranks** | xxxix |
| **For Reading to the Sick.**—Sowing and Reaping | xxxix |
| **Nurse Training in the Seventies.**—By E. D. | xl |
| **Appointments** | xl |
| **Everybody's Opinion.**—A Pro.'s Grievances—Private Nursing | xli |
| **An Old Prescription** | xli |
| **Notes and Queries** | xli |
| **Presentation** | xli |
| **The Invalid Children's Aid Association. II.**—What the Association Wants | xlii |
| **What to Read** | xlii |
| **Amusements and Relaxation** | xlii |

